# PIVKA-II level is correlated to development of portal vein tumor thrombus in patients with HBV-related hepatocellular carcinoma

**DOI:** 10.1186/s13027-019-0229-6

**Published:** 2019-05-14

**Authors:** Tao Li, Yuanzi Yu, Juan Liu, Xiangguo Tian, Meng Kong, Lei Wu, Shaocan Tang, Shengqing Gu, Jingfang Zhao, Yi Cui, Jinhua Hu

**Affiliations:** 1 Department of Gastroenterology, Provincial Hospital Affiliated to Shandong University, 324, Jing 5 Rd, Jinan, 250021 Shandong Province China; 2 Department of Gastrointestinal surgery, Provincial Hospital affiliated to Shandong University, Jing 5 Road, Jinan, People’s Republic of China; 3 Department of rehabilitation, Provincial Hospital affiliated to Shandong University, Jing 5 Road, Jinan, People’s Republic of China; 4 Department of Gastroenterology, Shouguang peoples’ Hospital, No.45, health street, Shouguang city, Weifang city, People’s Republic of China

**Keywords:** Hepatocellular carcinoma, PIVKA-II, Portal vein tumor thrombus

## Abstract

**Aim:**

To evaluate the correlation of serum PIVKA-II levels and development of portal vein tumor thrombus (PVTT) in hepatocellular carcinoma (HCC) patients.

**Methods:**

One hundred and twenty-three patients with newly diagnosed HCC were included in this study between March 2016 and October 2018. Thirty-five of these patients were detected with PVTT and all subjects were randomly divided to analysis group (*N* = 73) and validation (*N* = 50) group. Serum levels of PIVKA-II, laboratory tests including serum aspartate aminotransferase, total bilirubin, platelet count, albumin levels were demonstrated in all the patients. T-test, chi-squared test and logistic regression was used for analyzing data. Diagnostic efficiency and cut-off value of PIVKA-II in PVTT development of HCC patients were calculated using receiver operator curve (ROC) analysis.

**Results:**

Serum level of PIVKA-II in HCC patients with PVTT was significantly higher than that in HCC patients without PVTT (995.8 mAU/ml vs 94.87 mAU/ml; *P* = 0.003), as well as D-dimer levels (2.12 mg/L vs 0.56 mg/L *P* = 0.001). Univariate analysis showed that high serum D-dimer level was an independent risk factor for development of PVTT (OR = 1.22, 95%CI 1.02–1.45). ROC curve showed that among analysis group, the area under ROC curve (AUROC) of PIVKA-II was 0.73 (95%CI 0.59–0.86). For the detection of PVTT in HCC, PIVKA-II had a sensitivity of 83.7% and a specificity of 69.2% at a cutoff of 221.26 mAU/ml, which had a sensitivity of 85.71% and a specificity of 55.56% in validation group, respectively.

**Conclusion:**

Serum PIVKA-II level is a potential marker for diagnosis of PVTT in HCC patients, which may guide therapeutic strategy and assessment of tumor prognosis of HCC.

## Introduction

As the fifth most common cancer and the second highest cause of cancer-related death, hepatocellular carcinoma (HCC) is a severe health problem all over the world [1]. According to a recent research, more than one-third of HCC are diagnosed with portal vein tumor thrombosis (PVTT) or extrahepatic metastasis at BCLC C stage [2]. Prognosis of HCC patients with PVTT is especially poor, with a median survival of 3 months if no treatment is applied [3]. Macroscopic vascular invasion into the main portal veins or their branches can be detected by CT or MRI, while microscopic vascular invasion is identifiable only by microscopy [4]. Therefore, early diagnosis PVTT of HCC is of paramount importance for the treatment of HCC patients.

Prothrombin induced by vitamin K absence-II (PIVKA-II) is a prothrombin (PT) precursor with no coagulation activity secreted from HCC cells, and it has been shown to be a predictor of microvascular invasion (MVI) [5, 6]. Serum levels of PIVKA-II have been demonstrated significantly increased in HCC patients and employed as a diagnostic marker in Asia [6, 7]. Moreover, several studies have revealed that PIVKA-II serum levels were also significantly elevated in Eastern and European HCC patients [6, 8]. According to some in vivo studies, PIVKA-II could induce the overexpression of vascular endothelial growth factor (VEGF) and it may promote vascular invasion [9, 10]. Significantly higher PIVKA-II tissue expression was detected in HCC with microvascular invasion (MVI), as well as in serum [6]. Therefore, it could be employed as a predictor of microvascular invasion [6]. However, the role of PIVKA-II in the development of macrovascular invasion such as PVTT is not well demonstrated.

The present study aimed to detect the difference of PIVKA-II serum levels between HCC patients with PVTT and those without PVTT and evaluate the correlation between PIVKA-II and PVTT. In addition, we also investigated the correlation between serum levels of other HCC pathological characteristics such as AFP, D-Dimer and the development of PVTT.

## Patients and methods

### Patients

Between March 2016 and October 2018, a total of 123 consecutive patients with HBV-related HCC with or without PVTT were enrolled in this study. HCC diagnose was based on typical radiologic results of HCC on two dynamic image examinations or one dynamic technique with serum α-fetoprotein (AFP) level ≥ 200 ng/ml [11, 12]. Clinicopathologcial and laboratory data were obtained from all subjects. Imaging examination such as contrast-enhanced computed tomography (CECT) and magnetic resonance imaging (CEMRI) accompanied by intraoperative or postoperative histopathology were employed to diagnose PVTT in HCC patients [13]. A filling defect in the portal vein or its branch on contrast-enhanced computed tomography or magnetic resonance imaging were used to distinguish PVTT or thrombus. There were 4 types of PVTT based on the PVTT range in the portal vein: Type I, tumor thrombus in the segmental branches of the portal vein or above; Type II, tumor thrombus extending to the right or the left portal vein; Type III, tumor thrombus extending to the main portal vein; and Type IV, tumor thrombus extending to the main portal vein and the superior mesenteric vein [14].

The study was approved by ethical committee of Provincial Hospital affiliated to Shandong University. Written informed consent was obtained from each subject.

### Serum sample collection and assays

Peripheral blood samples were collected from each HCC patents right before surgeries or other treatments. The samples were spun and stored at − 80 °C until laboratory tests.

Serum level of PIVKA-II was detected with a Lumipulse G PIVKA-II reagent kit (FUJIREBIO Inc., Japan) on a LUMI-PULSE g1200 automatic immune analyzer according to manufacturer’s manual.

### Statistical analysis

Continuous variables with normal distribution are presented as mean ± standard deviation and were compared using Student’s *t* test. Categorical variables are expressed in absolute values and compared with the Chi-square test. Logistic Regression analysis was performed for independent variables of risk factors with PVTT in HCC patients, including gender, age, ALT, AST, PIVKA-II and D-dimer. Receiver operating characteristic (ROC) curve analysis was used to measure the diagnostic accuracy of PIVKA-II for the development of PVTT in HCC. All statistical analyses were accomplished using SPSS19.0 (IBM, Chicago, US). Statistical analysis was tested on two-sided settings and *P* < 0.05 considered as statistically significant.

## Results

### Baseline characteristics and comparison of PIVKA-II levels in analysis group and validation group

Thirty-five HCC patients with PVTT and 88 HCC patients without PVTT were enrolled in the present study between March 2016 and October 2018. All the subjects were randomly divided into the analysis group (*N* = 73) and validation group (*N* = 50). The baseline characteristics of all subjects are summarized in Table 1. The average age was 57.9 years, and 88.6% of the patients were male. Most HCC was accompanied with liver cirrhosis (85.4%). All patients were BCLC stage A, B or C due to multiple nodules, PVTT or extrahepatic metastasis. Baseline characteristics were comparable between the two groups (Table 1).

**Table 1:** Baseline characteristics of the study population

Variables	All patients	Analysis group	Validation group	*P* value
Sex F/M	14/109	9/64	5/45	0.69
Age (years)	57.94 ± 10.96	57.88 ± 10.03	58.02 ± 12.29	0.94
ALT (U/L)	30 (8–471.1)	27 (10–261)	35.5 (8–471.1)	0.30
AST (U/L)	47 (13–544)	38 (13–291)	52.5 (16–544)	0.06
TBiL (μmol/L)	18.3 (8.5–457)	17.8 (8.5–457.0)	20.0 (8.9–113.2)	0.41
ALB (g/L)	36.53 ± 4.91	36.98 ± 4.78	35.87 ± 5.06	0.22
D-Dimer (mg/L)	0.80 (0.10–15.61)	1.1 (0.27–15.61)	0.72 (0.10–13.95)	0.23
PTA (%)	80.62 ± 14.57	82.31 ± 12.92	78.14 ± 16.51	0.12
PLT (× 10^9^/L)	162.09 ± 99.10	161.96 ± 102.87	162.28 ± 94.37	0.97
AFP	441.06 (1.33–1210.0)	393.09 (1.33–1210.0)	511.09 (1.5–1210)	0.38
PIVKA-II (mAU/ml)	278.13 (1.6–75,000.00)	196.12 (12–75,000.00)	514.86 (1.6–75,000)	0.45
Liver cirrhosis				
YES/NO	105/18	30/5	75/13	0.95
Child-Pugh grade				
A	68	37	31	0.27
B	55	36	19	
PVTT, type				
I	34	21	13	0.91
II	51	30	21	
III	19	12	7	
IV	19	10	9	
BCLC stage				
A	12	9	3	0.48
B	76	43	33	
C	35	21	14	
HBsAg (COI)	4578 (12.2–8657.0)	4512 (12.2–7542.0)	5221.5 (35–8657.0)	0.38
HBeAg				
Positive	55	32	23	0.81
Negative	68	41	27	
HBV-DNA (IU/L)	7500.0 (0.0–4.16 × 10^7^)	5800.0 (0.0–4.16 × 10^7^)	21,750.0 (0.0–3.26 × 10^7^)	0.46

### HCC patients with PVTT were detected with higher D-dimer level in analysis group

**Fig. 1:**
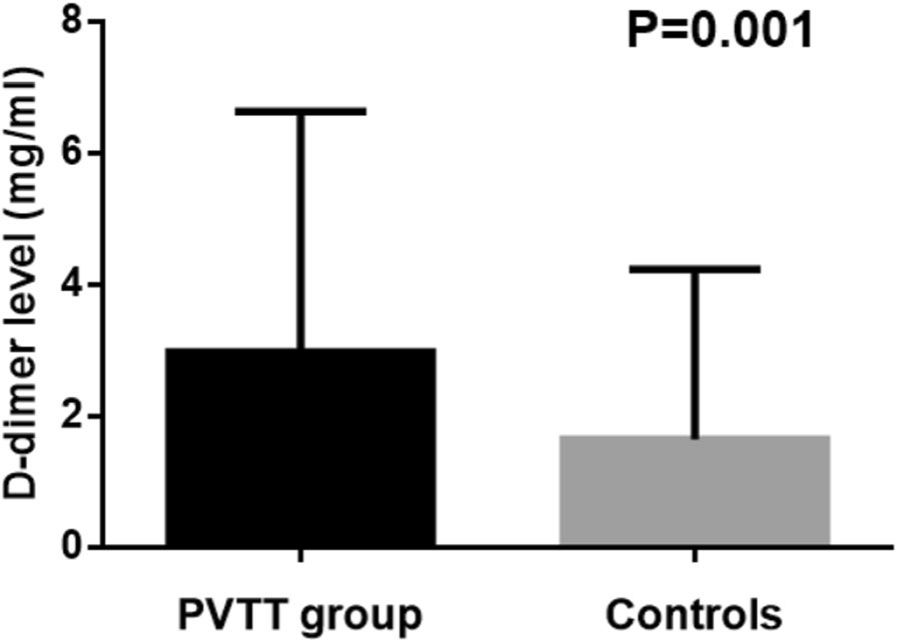
Difference of D-dimer plasma levels between HCC patients with PVTT and controls in analysis group. PVTT group:group of HCC patients with PVTT. Controls: group of HCC patients without PVTT

Elevated D-dimer level was detected in HCC patients with PVTT compared with those of HCC patients without PVTT, as shown in Table 2. The mean level of D-dimer among HCC patients with PVTT was 2.12(0.27–15.61) mg/ml, significantly higher than that of HCC patients without PVTT 0.56(0.10–13.95, *P* = 0.001) in analysis group (Fig. 1). Moreover, univariate analysis showed that high serum D-dimer level (OR = 1.22, 95%CI 1.02–1.45) was an independent risk factor for development of PVTT.

**Table 2:** Baseline characteristics of analysis group

Variables	Analysis group	HCC patients with PVTT	Controls	*P* value
Sex F/M	9/64	4/17	5/47	0.27
Age (years)	57.88 ± 10.03	55.33 ± 9.62	58.90 ± 10.10	0.17
ALT	27 (10–261.1)	39 (10–147)	24.5 (10–261.1)	0.97
AST	38 (13–291)	47 (13–105)	36.5 (17–291)	0.38
TBiL	17.8 (8.5–457)	22.4 (9.4–407.9)	17.35 (8.5–457.0)	0.26
ALB	36.98 ± 4.78	35.96 ± 4.27	37.39 ± 4.96	0.25
D-Dimer (mg/L)	1.1 (0.27–15.61)	2.12 (0.27–15.61)	0.56 (0.10–13.95)	0.001
PTA	82.31 ± 12.92	79.84 ± 11.34	83.31 ± 13.48	0.30
PLT (×10^9^/L)	161.96 ± 102.87	173.57 ± 118.47	157.27 ± 96.73	0.54
AFP	393.09 (1.33–1210.0)	469.17(3.10–1210)	362.36 (1.7–1210)	0.21
PIVKA-II (mAU/ml)	196.12 (12–75,000.00)	995.8 (12–75,000.00)	94.87 (13–70,997.0)	0.003
Child-Pugh class				
A	37	9	28	0.45
B	36	12	24	
HBsAg (COI)	4512 (12.2–7542.0)	5067.0 (62.33–7070.0)	4106 (12.2–7542.0)	0.35
HBeAg				
Positive	32	11	21	0.43
Negative	41	10	31	
HBV-DNA	5800.0 (0.0–4.16x10^7^)	4800.0 (0.0–2.99x10^6^)	6285.0 (0.0–4.16x10^7^)	0.81

### Diagnostic value of D-DIMER for PVTT in HCC patients of analysis group and validation group

A ROC curve was conducted to assess the diagnostic value of D-dimer for PVTT development in HCC patients, HCC patients without PVTT were enrolled as control in analysis group. To identify cutoff values that could best distinguish PVTT in HCC patients of analysis group, ROC curves were employed. The AUROC of D-dimer was 0.75 (95%CI 0.63–0.87, *P* = 0.001), However, AUROC of D-dimer in validation group was 0.57 (95%CI 0.39–0.75, *P* = 0.43).

### Elevated PIVKA-II level was detected in HCC patients with PVTT in analysis group

**Fig. 2:**
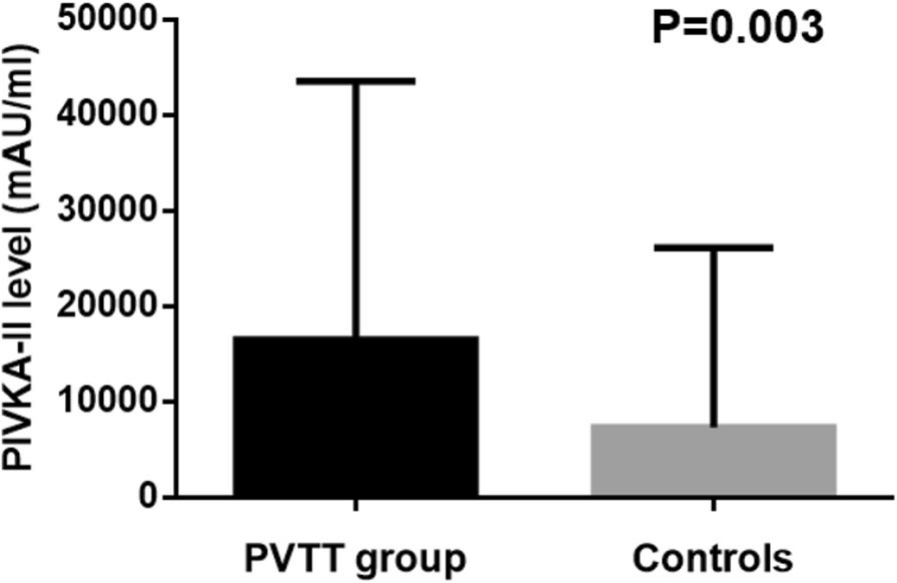
Difference of PIVKA-II plasma levels between HCC patients with PVTT and controls in analysis group. PVTT group:group of HCC patients with PVTT. Controls: group of HCC patients without PVTT

PIVKA-II level as well as other laboratory data of HCC patients with PVTT in analysis group were compared with those of HCC patients without PVTT, as shown in Table 2. The median level of PIVKA-II among HCC patients with PVTT was 995.80(12–75,000.00) mAU/ml, significantly higher than that of HCC patients without PVTT 94.87(13–75,000) mAU/ml with *P* = 0.003 (Fig. 2). Univariate analysis showed that high PIVKA-II level (OR = 1.95, 95%CI 1.23–3.09) was an independent risk factor for development of PVTT.

### Diagnostic value of PIVKA-II for PVTT in HCC patients of analysis group

**Fig. 3:**
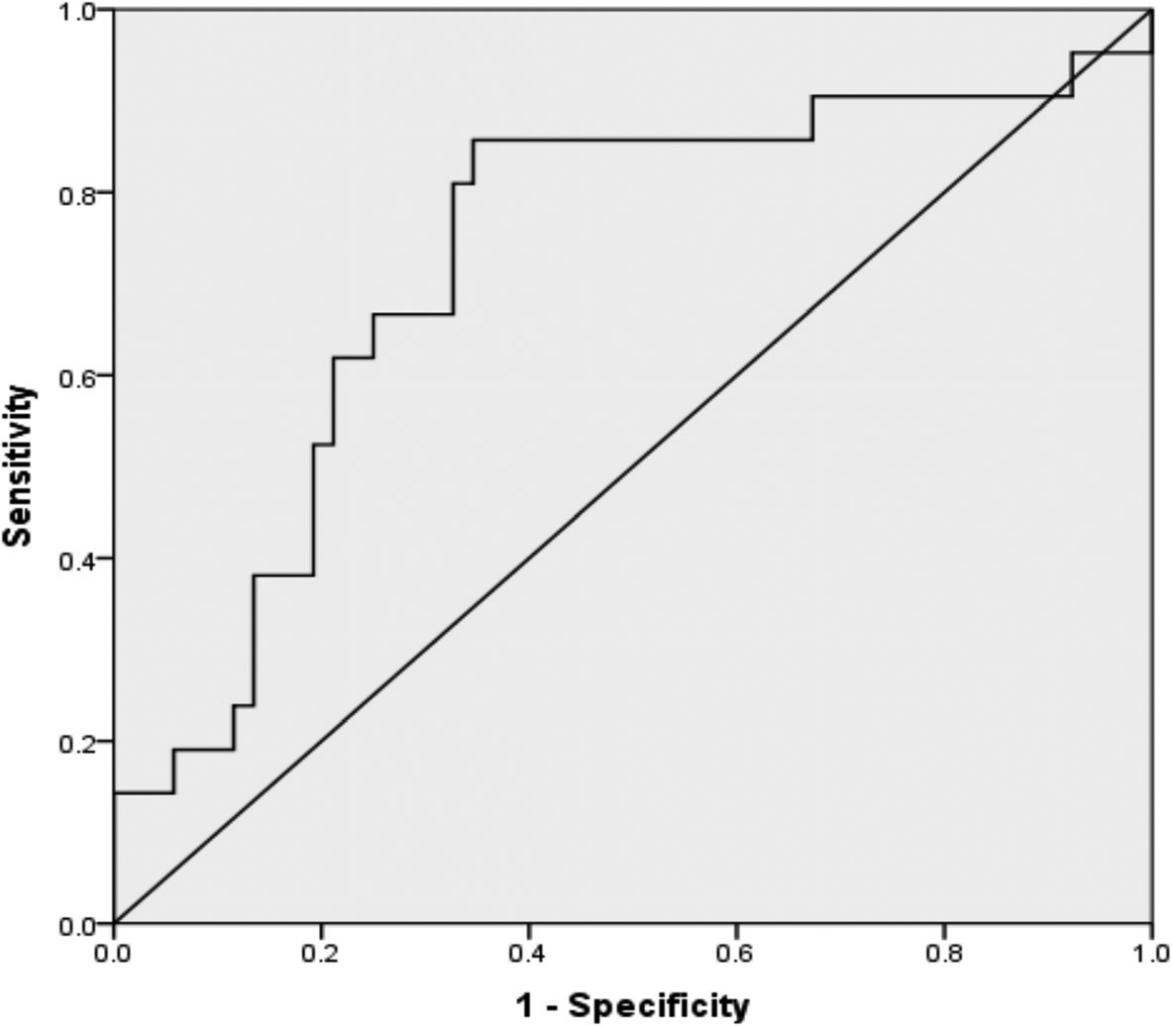
Diagnostic values of PIVKA-II in HCC patients with PVTT. The AUROC of PIVKA- II to diagnose HCC patients with PVTT was 0.70 (95%CI 0.60-0.81). For the diagnosis of PVTT in HCC, PIVKA-II had a sensitivity of 77.1% and a specificity of 63.6% at a cutoff of 327.69 mAU/ml.

A ROC curve was conducted to assess the diagnostic value of PIVKA-II for PVTT development in HCC patients, HCC patients without PVTT were enrolled as control for analysis. To identify cutoff values that could best distinguish PVTT in HCC patients from controls, ROC curves were plotted. The AUROC of PIVKA-II was 0.73 (95%CI 0.59–0.86, *P* = 0.003). The optimal cutoff value of PIVKA-II was 221.26 mAU/ml, with a sensitivity of 83.70% and a specificity of 69.20% (Fig. 3).

### Validation of PIVKA-II for PVTT detection in HCC patients

To validate the diagnostic value of PIVKA-II in development of PVTT in HCC patients, a ROC curve was conducted in validation group. ROC curves were plotted. The AUROC of PIVKA-II was 0.84 (95%CI 0.70–0.97, *P* < 0.01). The sensitivity and specificity of cutoff value 221.26 mAU/ml were 85.71 and 55.56%, respectively.

## Discussion

PVTT is an important predictor and prognostic factor for recurrence of HCC patients, which is occurred in about 10–40% patients with HCC at the time of diagnosis [15–17]. PVTT could increase portal venous pressure, decrease the blood flow to the liver, which can result in gastrointestinal hemorrhage or liver failure. Therefore, PVTT is a major factor to influence the median OS duration of postoperative HCC patients [15]. In the present study, we found that serum level of PIVKA-II was associated with the development of PVTT in HCC patients.

Up to date, PIVKA-II has been employed as an alternative tumor marker of AFP for HCC diagnose including early HCC, with a cut-off value of 40 mAU/ml [7, 18, 19]. The current study showed that PIVKA-II could also be a marker of PVTT in HCC patients. For that, HCC patients with PVTT demonstrate significantly higher PIVKA-II level compared with HCC patients without PVTT and the optimal cutoff value of PIVKA-II in diagnostic of PVTT in HCC patients was 221.26 mAU/ml, with a sensitivity of 83.70% and a specificity of 69.20%. In line with our findings, other researchers also found that serum PIVKA-II levels in HCC patients elevated in parallel with the progression of HCC, which are associated with poor prognosis [20]. Moreover, serum PIVKA-II levels in HCC patients significantly elevated according to BCLC stage, while AFP levels did not [21].

The present study indicated that serum PIVKA-II level could be employed as a biomarker for PVTT development HCC patients. In combine with other researches, we hypothesize that PIVKA-II may play a role in the development of PVTT in HCC patients along with the prognosis. According to a previous research, PIVKA-II could promote proliferation and migration of vascular endothelial cells, elevated serum level of PIVKA-II is associated with microvascular invasion [5]. For instance, Kim et al., reported that elevated serum PIVKA-II level was associated with microvascular invasion in small HCC (< 2 cm), and serum PIVKA-II level ≥ 200 mAU/mL was a predisposing factor for tumor recurrence after surgery [22]. Another recent meta-analysis study revealed that higher PIVKA-II could be a predictor of unfavorable overall survival in hepatocellular carcinoma patients receiving curative ablation [23]. Moreover, PIVKA-II has been employed as an important factor in consideration of liver transplantation for HCC patients [24].

Another interesting result in the current study indicated that D-Dimer was an independent risk factor for development of PVTT. Not only in PVTT patients, several studies revealed that high D-Dimer levels were also found in both liver cirrhosis and HCC patients [25]. In consistent with our findings, Liu, et al., also reported that D-dimer level elevated in advanced HCC patients and predicted poor prognosis [25, 26]. We hypothesize that thrombotic invasion of portal or hepatic vein may be involved in development of PVTT and result in high D-dimer level. D-dimer may serve as a complementary indicator that could detect the development of PVTT in HCC patients.

To our best knowledge, the present study shows for the first time that PIVKA is useful for predicting the risk of PVTT. Further studies are necessary to validate this correlation.
